# Cervical sympathetic chain schwannoma masquerading as a vagus nerve: a case report

**DOI:** 10.1093/jscr/rjac228

**Published:** 2022-05-22

**Authors:** Azeddine Lachkar, Faycal Roubi, Drissia Benfadil, Fahd Elayoubi

**Affiliations:** ENT and Head and Neck Surgery Department, University Hospital Center Mohammed VI, Faculty of Medicine and Pharmacy, Mohammed First University, Oujda, Morocco; ENT and Head and Neck Surgery Department, University Hospital Center Mohammed VI, Faculty of Medicine and Pharmacy, Mohammed First University, Oujda, Morocco; ENT and Head and Neck Surgery Department, University Hospital Center Mohammed VI, Faculty of Medicine and Pharmacy, Mohammed First University, Oujda, Morocco; ENT and Head and Neck Surgery Department, University Hospital Center Mohammed VI, Faculty of Medicine and Pharmacy, Mohammed First University, Oujda, Morocco

**Keywords:** schwannoma, cervical sympathetic chain, vagus nerve, Horner’s syndrome

## Abstract

Schwannoma arising from the cervical sympathetic chain are rare slow-growing tumors which represent a diagnostic challenge. We report a 80-year-old female patient presented with anterior neck triangle swelling. The radiological assessment was based on computed tomography and magnetic resonance imaging, which led to a preoperative diagnosis of vagus nerve schwannoma. However, surgical treatment revealed a cervical sympathetic chain mass rather than a vagus nerve mass. A complete removal was performed, and the anatomopathological examination was in favor of a schwannoma. In post-operative state, the patient presented a well-tolerated Horner’s syndrome.

## INTRODUCTION

Schwannoma is a benign mesenchymal tumor developed exclusively from Schwann’s sheath cells that surround nerve fibers of the peripheral nervous system. Schwannomas are found in 25% of cases at the cervical level, most often at the expense of the vagus nerve. The other localizations are rarer in particular the schwannoma of the cervical sympathetic chain [1]. We report an unusual cause of neck swelling arising from cervical sympathetic chain which was misdiagnosed preoperatively as a vagus nerve schwannoma.

## CASE PRESENTATION

Our patient was a 80-year-old female, who had no medical history and consulted for a left cervical mass progressively evolving over 3 years and associated with dysphagia without dysphonia or dyspnea. Physical examination revealed a left laterocervical swelling measuring 6 × 5cm in size. It was firm, smooth, regular in margins and pulsatile. No nerve palsies noted.

**Figure 1:**
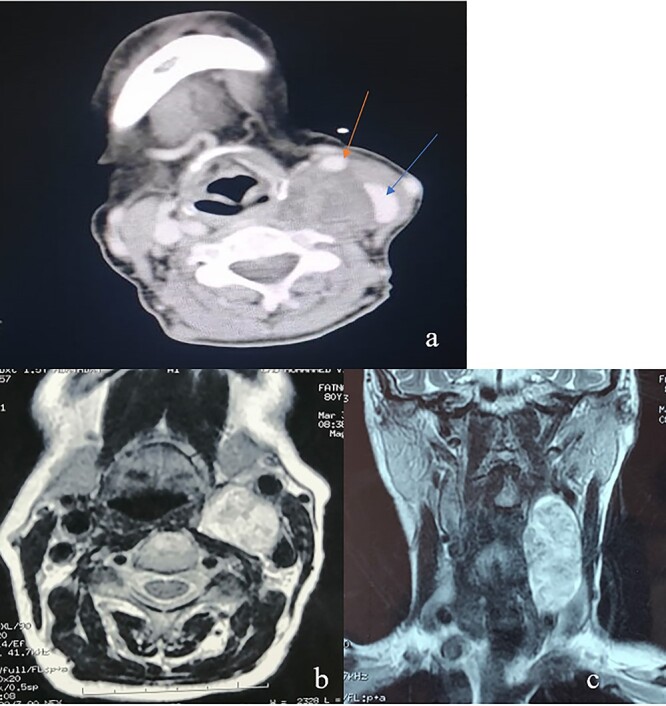
(**a**) An axial CT of the neck revealing an oval heterogeneous mass in the left carotid space measuring 37 × 30 × 57 mm; the IJV (blue arrow) is displaced laterally and the CCA (red arrow) is displaced anteriorly; this is often a characteristic sign of VNS; (**b**) an axial T2-weighted MRI; left carotid space mass, noninfiltrating widening the space between CCE and IJV; (**c**) an coronal T2-weighted MRI showing a left carotid space mass with vertical major axis.

Computed tomography (CT) scan of the neck described a left neck mass (3.7, 3, 5.7 cm), hypodense, heterogeneous and not enhanced after injection of the contrast product. The mass displaced the common carotid artery (CCA), the external carotid artery (ECA) as well as the internal carotid artery (ICA) anteriorly and the internal jugular vein (IJV) anterolaterally. Magnetic resonance imaging (MRI) showed a low signal intensity T1-weighted and a high signal intensity T2-weighted mass (Fig. 1). These radiological findings aligned most closely with a diagnosis of vagal nerve schwannoma (VNS), therefore cervical sympathetic chain schwannoma (CSCS) could not be excluded.

**Figure 2:**
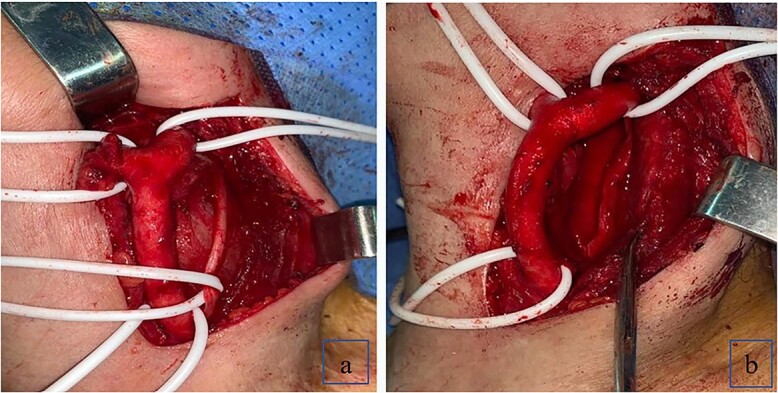
(**a**) Schwannoma causing anterior displacement of CCA, ICA and ECA; the vagus nerve running over the top of the mass; (**b**) complete resection of the schwannoma with preservation of the vagus nerve.

**Figure 3:**
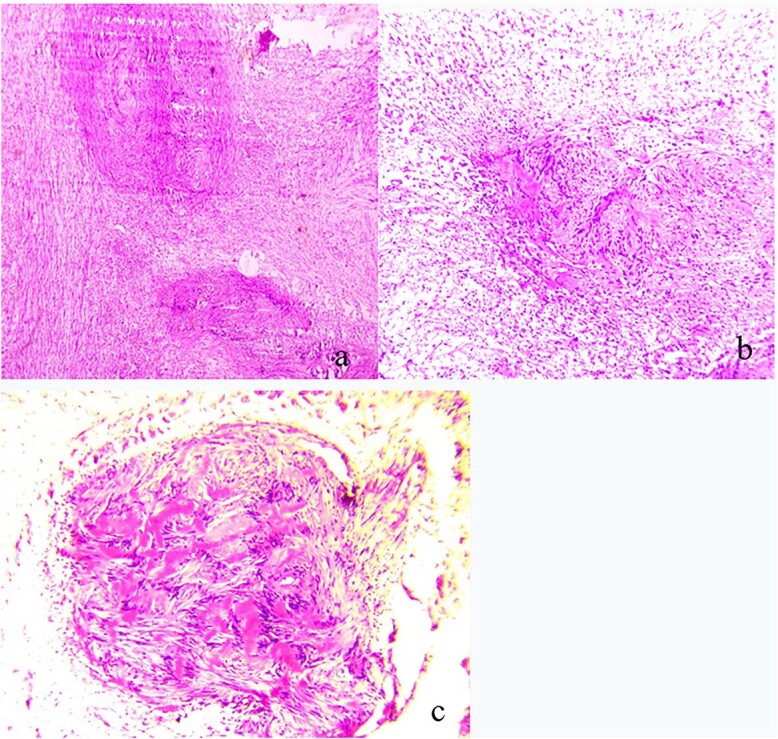
(**a**) Pathologic findings demonstrating plexiform schwannoma; (**b**) both Antoni A and Antoni B areas; (**c**) Verocay bodies.

The patient underwent surgical excision with a transcervical approach. Intra-operatively, the mass was found lying medial to the CCA, the ICA and the ECA. The vagus nerve was visualized running over the top of the mass, uninvolved in the tumor formation (Fig. 2). Instead, the cervical sympathetic chain was identified superiorly and inferiorly to the mass, making CSCS the most likely diagnosis. Resection was performed with dissection and preservation of the vagus nerve. The Horner’s syndrome was observed in the left eye after surgical excision. Pathology reports showed plexiform schwannoma, including Antoni A and Antoni B areas with Verocay bodies (Fig. 3).

## DISCUSSION

CSCS is an unusual diagnosis. Although the schwannoma in this case atypically developed in the midcervical portion of the carotid space, cervical schwannomas are commonly reported in the parapharyngeal space. The vagus nerve is the most common nerve of origin [2].

Schwannomas are typically seen in patients between the ages of 30 and 70, with a sex ratio of 1. The diagnosis of a schwannoma is difficult not only because of the wide differential diagnosis of cervical mass but also because of the rarity and nonspecific clinical presentation, usually presenting as an asymptomatic, slow-growing mass. A CSCS is more easily evoked when the cervical mass is associated with a Horner's Syndrome by compression of the cervical sympathetic nerve. However, the latter is rarely found at the time of diagnosis [3]. Indeed, unlike the neurofibroma, the schwannoma is an encapsulated tumor that does not infiltrate the perineural nerve sheath and the nerve filaments. The pulsatile nature of the mass may be due to hyper-vascularization of the schwannoma or due to the reflection of the carotid system which may be pushed forward. Imaging with CT and MRI is the preferred diagnostic modality for evaluating cervical schwannomas. The absence of a gap between the carotid artery and IJV combined with an anterior displacement of both vessels on imaging frequently occurs in CSCS, while the schwannoma originating from the vagus nerve tends to widen the space between the internal carotid and the IJV [4]. An uncertain nerve of origin makes informing the patient of specific surgical outcomes difficult. A medially displaced carotid artery was found to be more determinate of VNS, while a laterally displaced carotid artery was found to be more commonly associated with CSCS. In addition, peripheral enhancement of the mass on T2-weighted MRI was significantly associated with VNS, while homogenous enhancement was found in CSCS [5].

Surgical excision is the preferred treatment for these tumors. In most cases, extra-capsular resection is performed with resultant nerve sacrifice. Intracapsular enucleation, as an alternative approach, has been shown to result in less morbidity due to nerve preservation but is frequently difficult to achieve due to the intimate contact with CSC and dense attachment of the tumor. Our patient developed a well-tolerated partial Horner’s syndrome post-resection, which is the most common sequelae associated with extracapsular excision of the tumor [6].

## CONCLUSION

This case presents a rare case of a CSCS masquerading as a VNS. This case highlights recently enhanced diagnostic criteria to improve preoperative accuracy and unusual post-operative sequalae.

## Data Availability

The patient’s data are available upon reasonable request to the corresponding author.
